# Origin and neurochemical properties of bulbospinal neurons projecting to the rat lumbar spinal cord via the medial longitudinal fasciculus and caudal ventrolateral medulla

**DOI:** 10.3389/fncir.2014.00040

**Published:** 2014-04-28

**Authors:** Zilli Huma, Amy Du Beau, Christina Brown, David J. Maxwell

**Affiliations:** Spinal Cord Group, Institute of Neuroscience and Psychology, College of Medicine, Veterinary Medicine and Life Sciences, University of GlasgowGlasgow, UK

**Keywords:** brainstem, spinal cord, descending system, neurotransmitters, motor control, tract-tracing, confocal microscopy, neuroanatomy

## Abstract

Bulbospinal systems (BS) originate from various regions of the brainstem and influence spinal neurons by classical synaptic and modulatory mechanisms. Our aim was to determine the brainstem locations of cells of origin of BS pathways passing through the medial longitudinal fasciculus (MLF) and the caudal ventrolateral medulla (CVLM). We also examined the transmitter content of spinal terminations of the CVLM pathway. Six adult rats received Fluorogold (FG) injections to the right intermediate gray matter of the lumbar cord (L1–L2) and the b-subunit of cholera toxin (CTb) was injected either into the MLF or the right CVLM (3 animals each). Double-labeled cells were identified within brainstem structures with confocal microscopy and mapped onto brainstem diagrams. An additional 3 rats were injected with CTb in the CVLM to label axon terminals in the lumbar spinal cord. Double-labeled cells projecting via the MLF or CVLM were found principally in reticular regions of the medulla and pons but small numbers of cells were also located within the midbrain. CVLM projections to the lumbar cord were almost exclusively ipsilateral and concentrated within the intermediate gray matter. Most (62%) of terminals were immunoreactive for the vesicular glutamate transporter 2 while 23% contained the vesicular GABA transporter. The inhibitory subpopulation was glycinergic, GABAergic or contained both transmitters. The proportions of excitatory and inhibitory axons projecting via the CVLM to the lumbar cord are similar to those projecting via the MLF. Unlike the MLF pathway, CVLM projections are predominantly ipsilateral and concentrated within intermediate gray but do not extend into motor nuclei or laminia VIII. Terminations of the CVLM pathway are located in a region of the gray matter that is rich in premotor interneurons; thus its primary function may be to coordinate activity of premotor networks.

## Introduction

Bulbospinal (BS) systems are composed of heterogeneous pathways that originate from the brainstem. They influence a variety of spinal networks including those concerned with motor control, sensory input (including pain) and autonomic function. It is likely that one of the functions of BS systems is to coordinate the activity of spinal networks involved in these various processes (Holstege, 1996; Hardy et al., 1998; Tavares and Lima, 2002). The neurons that give rise to BS pathways project to the spinal cord via fiber tracts in the medulla (Mitani et al., 1988). These tracts include the MLF, which contains axons principally involved in motor control (Jankowska et al., 2007; Jankowska, 2008) and axonal systems within the caudal ventrolateral medulla (CVLM) which have a role in sensory and autonomic control in addition to an influence on motor activity (Tavares and Lima, 2002). Some classes of bulbospinal neuron which arise principally from the medullary reticular formation are responsible for conveying signals from motor centers in the brain which include the mesencephalic locomotor center and the primary motor cortex (Matsuyama et al., 2004; Jordan et al., 2008). These neurons have complex effects on motor function; they are not only involved in the maintenance of posture but may also have a role in goal-directed activities such as reaching (Drew et al., 2004; Riddle et al., 2009). The descending axons of these cells innervate wide areas of the spinal gray matter and individual axons give rise to collaterals that terminate at many segmental levels (Peterson et al., 1975; Matsuyama et al., 1999; Reed et al., 2008). Therefore an individual BS cell has the capacity to influence a wide range of spinal neurons and, on this basis, it has been suggested that these neurons are components of a system that is responsible for integrating “common neuronal elements” in order to produce a variety of coordinated motor patterns (Drew et al., 2004; Matsuyama et al., 2004). Although the action of BS neurons on their spinal targets is predominantly excitatory (Jankowska et al., 2007; Jankowska, 2008) immunocytochemical evidence shows that some BS axons also contain inhibitory neurotransmitters (Holstege, 1996; Du Beau et al., 2012; Hossaini et al., 2012). Therefore these systems can have monosynaptic inhibitory actions on spinal neurons in addition to direct excitatory actions. Such actions could serve to coordinate motor output by facilitating or depressing specific components of motor networks.

During a series of experiments to label spinal cells projecting to the lateral reticular nucleus we observed anterogradely labeled terminals in the lumbar spinal cord that had ipsilateral projections and terminated predominantly in the intermediate gray matter. Although these injections were focused upon the CVLM it was not clear if this was the origin of this pathway as the b subunit of cholera toxin (CTb) we used to label descending axons is taken up by axons of passage in addition to cell bodies and axon terminals (Chen and Aston-Jones, 1995). Electrical stimulation of the CVLM can evoke responses in spinal neurons (Tavares and Lima, 2002) but electrical stimulation not only activates cells but also axons of passage and therefore the neurons that mediate these effects may be located at some distance from the site of stimulation. Although a number of studies have documented the origins of BS cells within structures of the rat brainstem (Leong et al., 1984; Zemlan et al., 1984; Rye et al., 1988; Reed et al., 2008; Hossaini et al., 2012), the exact locations of cells with axons that project via the MLF are still largely unknown. Furthermore the locations of cells that give rise to the pathways passing through the CVLM are completely unknown. In view of the limited anatomical information available concerning these systems, our primary aim was to determine the locations of cells that give rise to these pathways. We exploited the propensity for CTb to be taken up by axons of passage by injecting it into MLF or the CVLM and, in the same experiments, we injected Fluorogold (FG) into the spinal cord. Hence we were able to map the locations of double-labeled cells in the brainstem that project to the spinal cord via these two routes.

Bulbospinal systems influence spinal neurons by means of classical synaptic mechanisms but also have a modulatory function which can be direct or indirect via spinal interneurons (e.g., see Jordan et al., 2008). A variety of neurotransmitters and neuromodulators have been associated with these pathways including glutamate, GABA, glycine, monoamines, and peptides. In a previous study (Du Beau et al., 2012) we investigated transmitter phenotypes of lumbar spinal terminations of the BS systems that passes through the MLF. Stereotaxic injections of CTb within the MLF revealed terminals in the lumbar spinal cord that were concentrated within the intermediate gray matter and the ventral horn. Although the majority (59%) of axon terminals in the lumbar spinal cord were glutamatergic, a sizable minority were inhibitory (20%) and these could be subdivided into those that are GABAergic (7%), those that are glycinergic (9%) and those that contained both transmitters (3%). None of the terminals contained serotonin and there was also a significant population (18%) that did not show immunoreactivity for any of the transmitters tested. A secondary aim of the study therefore was to determine the types of neurotransmitters associated with the CVLM pathway.

## Materials and methods

### Surgical procedures

In these experiments the MLF or CVLM was injected with the b subunit of cholera toxin (Sigma-Aldrich, Co., Poole, UK) which is a retrograde and anterograde tracer and the spinal cord was injected with Fluorogold (Fluorochrome, LLC, USA) which is a retrograde tracer that is primarily taken up by axon terminals. All animal procedures were carried out according to British Home Office legislation and were approved by the Glasgow University Ethical Review Committee. Nine adult male Sprague Dawley rats (Harlan, Bicester, UK) weighing between 250 and 350 g were anesthetized with isoflurane (up to 4% in oxygen), placed in a stereotaxic frame and maintained under deep anaesthesia. The skin at the back of the head was cut in the midline to expose the skull and a small burr hole was then made. The stereotaxic coordinates for injections are given in Table 1 (see Paxinos and Watson, 2005). A glass micropipette with a tip diameter of 20 μm filled with 1% CTb in distilled water was aligned with the burr hole and inserted into the brain. CTb (200 nl) was injected by pressure with a Pico-Injector (10 ms pulses at 20 psi; World Precision Instruments, Sarasota, USA) into the right MLF (3 animals) or the right CVLM (6 animals: 3 for spinal injections). At the conclusion of surgery, the scalp was sutured and animals were placed in an incubator to assist recovery.

**Table 1:** **Interaural stereotaxic coordinates used to target the medial longitudinal fascicle (MLF) and caudal ventrolateral medulla (CVLM) From Paxinos and Watson (2005)**.

	**Anterior-posterior**	**Medial-lateral**	**Dorsal-ventral**
MLF	−3.8 mm	−0.1 mm	+0.8 mm
CVLM	−4.8 mm	−1.8 mm	−0.4 mm

Following a period of 48 h, six animals (three MLF and three CVLM) were re-anesthetized with isoflurane and placed in a spinal frame. The thirteenth thoracic vertebra was identified according to the location of the last rib and a small dorsal midline incision was made at this level. A hole with a diameter of 1 mm was made adjacent to the midline in the laminar surface of the caudal part of the Th13 or L1 vertebrae to expose the dorsal surface of L1 or L2 segments of the spinal cord. Unilateral spinal injections of 50 nl were made with glass micropipettes containing 4% FG in distilled water. The tip of the injection pipette (20 μm in diameter) was inserted into the spinal cord to a depth of up to 1.5 mm from the surface at an angle of 15° to target the intermediate gray matter of the right side of the spinal cord. The wound was sutured and animals recovered uneventfully.

### Perfusion and fixation

Following a 7 day survival period from initial brain injections, rats were anaesthetized with pentobarbitone (1 ml i.p.) and perfused through the left ventricle with mammalian Ringer's solution followed by one litre of a fixative containing 4% formaldehyde in 0.1 M phosphate buffer (PB; pH 7.4) at room temperature. Spinal cords and brains were removed and post-fixed for 8 h at 4°C and were cut into 60 μm thick transverse sections with a Vibratome (Oxford Instruments, Technical Products International, Inc., USA). All sections were treated with an aqueous solution of 50% ethanol for 30 min to aid complete antibody penetration.

### Tissue processing

In experiments to examine spinally projecting cells via the MLF and CVLM, the brainstem was divided into the medulla, pons and midbrain by using a razor blade to cut the medullary-pontine junction in the coronal plane to separate the medulla and pons and similarly to separate the pons from the midbrain by cutting at a level just inferior to the inferior colliculi. Sections from these three regions were reacted with solutions of primary antibodies to identify CTb and FG for 48 h (See Table 2A for details). Subsequently they were incubated in secondary antibodies coupled to fluorphores for 3 h and mounted on glass slides with anti-fade medium, (Vectashield; Vector Laboratories, Peterborough, UK). Spinal injection sites containing FG were examined with UV epifluorescence and photographed whereas brainstem injection sites were visualized by using 3,3′-diaminobenzidine (DAB) as a chromogen. Sections were incubated in goat anti-CTb for 48 h followed by biotinylated anti-goat IgG for 3 h at room temperature. They were then incubated in avidin-horseradish peroxidase (HRP) for 1 h and hydrogen peroxide plus DAB was applied for a period of approximately 15 min to reveal immunoreactivity.

**Table 2:** **Antibodies used in the study**.

	**Primary antibody combination**	**Primary antibody concentration**	**Supplier**	**Secondary antibodies**
A	gt. CTb	1:5000	List Biological Laboratories, Campell, CA	Rh.Red
	rbt FG	1:5000	Chemicon/Millipore,CA,USA	Alexa488
B	mo. CTb	1:250	A. Wikström, University of Gothenburg	Rh.Red
	gp VGLUT1	1:5000	Millipore, Harlow, UK	Alexa488
	gp VGLUT2	1:5000	Millipore, Harlow, UK	Dylight 649
C	mo. CTb	1:250	A. Wikström, University of Gothenburg	Rh.Red
	rbt VGAT	1:1000	Synaptic System, Göttingen, Germany	Alexa488
	gp VGLUT1	1:5000	Millipore, Harlow, UK	Dylight 649
	gp VGLUT2	1:5000	Millipore, Harlow, UK	Dylight 649
D	gt. CTb	1:5000	List Biological Laboratories, Campell, CA	Rh.Red
	rbt GLYT2	1:1000	Millipore, Harlow, UK	Alexa488
	mo GAD67	1:1000	Millipore, Harlow, UK	Dylight 649
E	mo. CTb	1:250	A. Wikström, University of Gothenburg	Rh.Red
	rbt Serotonin	1:100	Affiniti, Exeter, UK	Alexa488

*gt, goat; rbt, rabbit; mo, mouse; gp, guinea pig; FG, Fluorogold; CTb, the b subunit of cholera toxin; VGLUT1, vesicular glutamate transporter 1; VGLUT2, vesicular glutamate transporter 2; VGAT, vesicular GABA transporter; GLYT2, glycine transporter 2; GAD67, 67 isoform of glutamate decarboxylase. In Experiment C tissue was incubated in a mixture of VGLUT1+2 antibodies*.

In experiments to examine descending axons, brainstem injection sites were processed as described above. Spinal sections from L3–5 segments were incubated in a combination of antibodies (see Tables 2B–E for details) against: (1) CTb, VGLUT1, and VGLUT2; (2) CTb, a mixture of VGLUT1+2 antibodies and VGAT; (3) CTb, glutamic acid decarboxylase 67 (GAD67) and the glycine transporter 2 (GLYT2) or (4) CTb and serotonin (5-HT). Thereafter the sections were washed in PBS, incubated in secondary antibodies coupled to fluorphores for 3 h and had a final wash with PBS before they were mounted on glass slides.

### Data acquisition and analysis

To document the locations of double-labeled cells in the brainstem, transmitted light images of all incubated sections were captured digitally using a x1 lens (AxioVision 4.8 software Carl Zeiss, Inc, Germany). These sections were used to identify the various levels of the brainstem according to the rat brain atlas of Paxinos and Watson (2005). The sections were scanned and montaged using a confocal microscope (LSM 710, Zeiss, Germany: magnification 20× lens, zoom factor 0.6 at z-steps of 1 μm intervals). Spinally projecting cells were identified by the presence of FG whereas cells with axons passing through the MLF or CVLM appeared red and cells containing both FG and CTb were yellow. Scans of entire brainstem sections at representative levels (approximately Bregma anterior-posterior levels: −14; −13; −12; −9; −8; −6) were taken. Confocal images were analyzed with Neurolucida for Confocal software (Microbrightfield Inc, Colchester, VT, USA) in order to estimate numbers of cells contained within brainstem structures. A counting grid (100 × 100 μm) was applied to each section and cells were counted within 2–5 adjacent sections (depending on the size of the structure) from each brainstem level for the two groups of animals. Images were exported to Adobe Photoshop so that locations of cells could be plotted onto outline diagrams of the brainstem (Paxinos and Watson, 2005). Cell counts are presented as averaged numbers of double-labeled cells per structure for the three animals in each group. This was done for cells contralateral and ipsilateral to spinal injections with the exception of cells within midline structures such as raphe nuclei or the MLF where data on both sides were pooled.

For axons labeled by CVLM injections, confocal microscope images were acquired from a minimum of six sections per animal. Fields containing CTb-labeled axon terminals were scanned by using a x40 oil-immersion lens with a zoom factor of 2 at 0.5 μm intervals. For each section three fields with a 100 × 100 μm scanning area were obtained from different regions of the gray matter. Stacks of images were analyzed with Neurolucida for Confocal software (MBF Bioscience, Colchester, VT, USA). Image stacks were initially viewed so that only CTb immunoreactivity was visible. All CTb labeled terminals within the scanning box from each animal were used for analysis. The terminals were then examined in the blue and green channels in order to assess expression of transmitter-related markers. The percentage of double-labeled CTb terminals as a proportion of the total number of CTb terminals was calculated for each animal. This value was averaged for the three animals and expressed as the mean percentage ± the standard deviation (SD).

## Results

### Injection sites for double-labeling experiments

**Figure 1:**
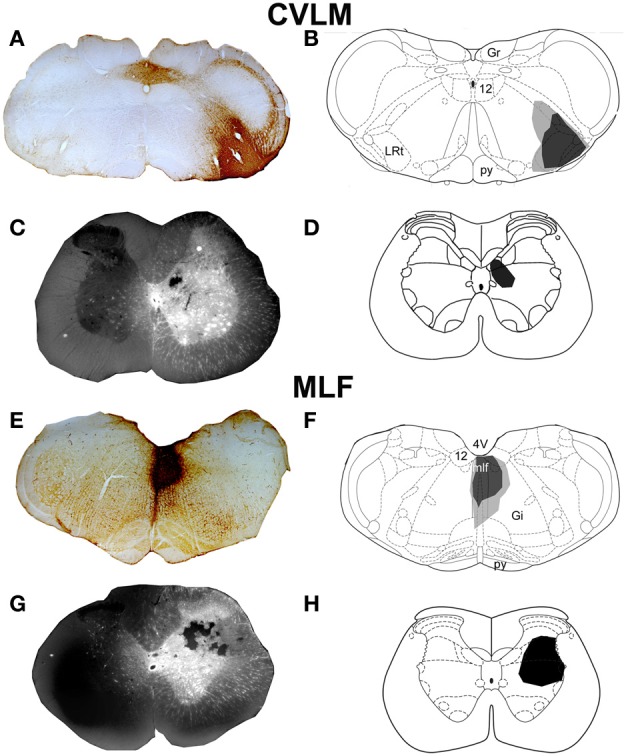
**CVLM and MLF injection sites with associated spinal injection sites. (A–D)** Shows an experiment to double-label cells with axons passing through the CVLM and **(E–H)** are taken from an experiment to label axons of cells passing through the MLF. **(A,E)** Show CTb injection sites in coronal sections of the medulla. **(B,F)** Show injection sites mapped onto outline diagrams (Paxinos and Watson, 2005). Black, the core and gray, spread of CTb. **(C,G)** Fluorescent images superimposed upon dark field images of the same sections illustrating Fluorogold injection sites at L1–2. Drawings showing the locations of spinal injections (black) are shown in **(D,H)**. Note the spread of Fluorogold within the gray matter surrounding the injection. 4V, 4th ventricle; 12, hypoglossal nucleus; CVLM, caudal ventrolateral medulla; Gi, gigantocellular reticular nucleus; Gr, gracile nucleus; LRt, lateral reticular nucleus; MLF, medial longitudinal fascicle; py, pyramid.

Representative examples of injection sites are shown in Figure 1. MLF injections were centered upon the right medial longitudinal fascicle. We attempted to minimize the extent of MLF injections but there was always some spread of CTb into the contralateral MLF. In addition there was also spread into the raphe obscuris, paramedian reticular nucleus and tectospinal tract. The rostro-caudal spread of these injections was ±1.38 mm on average from the location of the injection. CVLM injections were centered upon the lateral reticular nucleus but spread into surrounding structures including the parvicellular reticular nucleus, the internal reticular nucleus and the nucleus ambiguous; however they did not encroach on the rubrospinal tract. The rostro-caudal spread of CTb in CVLM injections extended over a distance of ±0.65 mm on average. Spinal injections were confined to L1–2 and injection sites were present in the intermediate gray matter in all experiments but the precise location of each injection varied. Considerable spread of Fluorogold was observed within the gray matter ipsilateral to injection sites but there was no spread to the contralateral gray matter. The rostro-caudal spread of FG within the spinal gray matter was ±0.27 mm on average on each side of the injection site.

### Distribution of cells in brainstem

**Figure 2:**
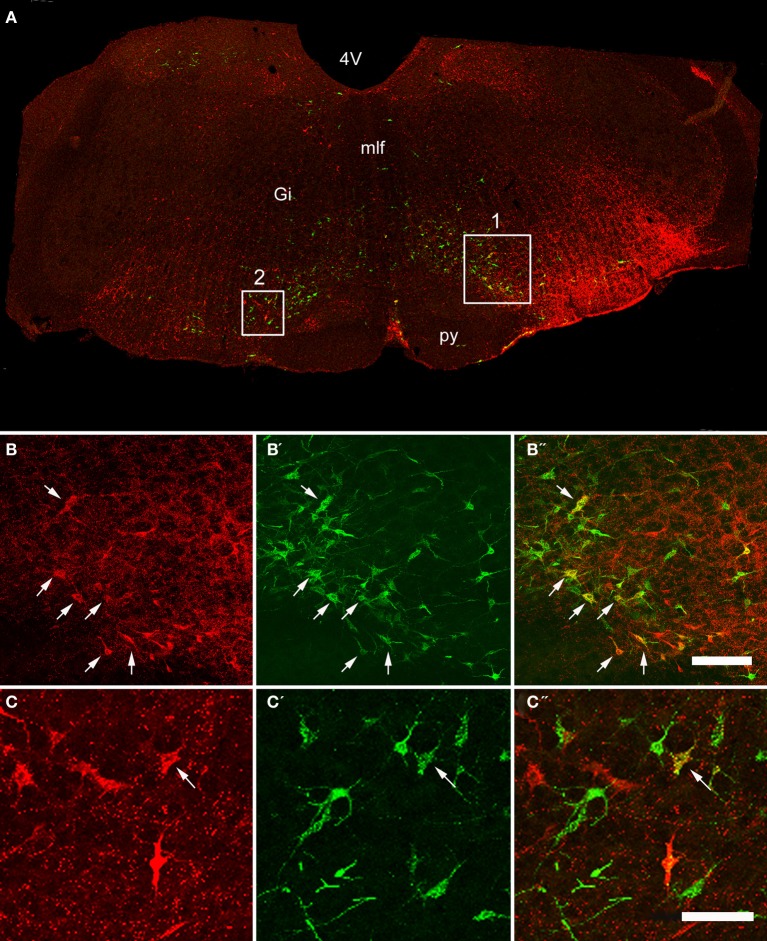
**Confocal images of double-labeled cells in the Medulla. (A)** Shows a montage of a coronal section through the medulla of an animal that had received a CVLM and spinal injection, (both on the right side). Areas 1 and 2 within the boxes are shown at higher magnification in series **(B,C)** respectively which are short projected sequences of confocal images showing cells labeled from the CVLM **(B,C)**, the spinal cord **(B′,C′)** and merged images showing yellow double-labeled cells **(B″,C″)**. Arrows indicate Double-labeled cells. 4V, 4th ventricle, Gi, gigantocellular reticular nucleus, mlf, medial longitudinal fasciculus, py, pyramid. Scale bars = 100 μm.

**Figure 3:**
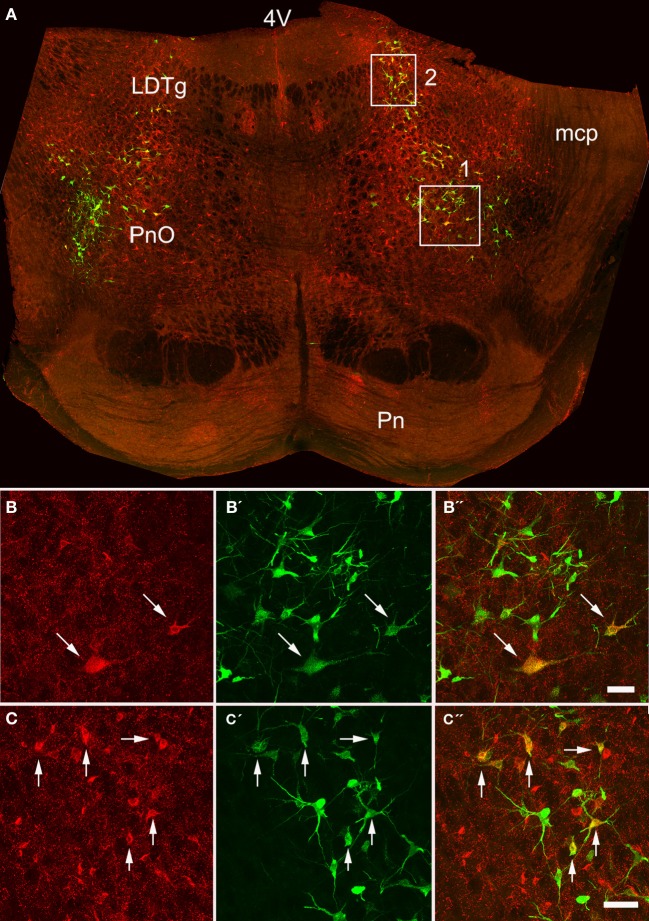
**Confocal images of double-labeled cells in the pons. (A)** Shows a montage of a coronal section through the pons of an animal that had received a MLF and spinal injection, (both on the right side). Areas 1 and 2 within the boxes are shown at higher magnification in series **(B,C)** respectively which are short projected sequences of confocal images showing cells labeled from the MLF **(B,C)**, the spinal cord **(B′,C′)** and merged images showing yellow double-labeled cells **(B″,C″)**. 4V, 4th ventricle; LDTg, laterodorsal tegmental nucleus, mcp, middle cerebellar peduncle, Pn, pontine nuclei, PnO, pontine reticular nucleus, oral part. Arrows indicate Double- labeled cells. Scale bars = 50 μm.

Double-labeled cells were found within the medulla and pons for CVLM and MLF injections (Figures 2, 3) and small numbers of additional cells were found in the midbrain (69 out of a total of 2033 cells). For MLF injections the average percentage (±SD) of double-labeled cells found in the medulla for the three animals was 68 ± 5.9% (494/722 cells in total for all 3 animals) and the equivalent value for CVLM injections was 84 ± 10.2% (1040/1242 cells in total). In each experiment there was some variation in the distribution of cells as a consequence of inevitable differences in brainstem and spinal injections but consistencies in the distribution of cells were apparent across all animals in each experimental group. A full list of all structures containing double-labeled cells (average number per structure) is given in Table 3.

**Table 3:** **Location of double-labeled cells in the brainstem following Fluorogold injections into the right lumbar cord and injections of cholera toxin in the MLF or right CVLM**.

	**MLF**		**CVLM**	
	**IPSI**	**CONTRA**	**IPSI**	**CONTRA**
**MEDULLA**				
Gi	69.7	6.7	52.7	41.7
IO	7.7	18.3	37.7	20.0
LPGi	0.0	1.0	24.7	12.7
MdV	7.3	0.0	22.0	8.3
Mlf	17.0		16.3	
5n	0.0	0.0	4.3	4.3
7n	0.0	0.0	6.0	2.7
RMg	14.3		13.3	
Rob	3.3		14.3	
RPa	0.0		20.0	
RR	2.7	0.0	0.0	0.0
Cu	0.0	0.0	0.0	0.7
LPAG	0.0	0.0	0.3	0.0
LRt	0.0	0.0	5.3	0.0
MVe	9.3	0.0	3.0	5.0
PPy	0.0	0.0	1.3	1.3
Sol	0.0	6.3	0.0	4.7
SPO	0.0	0.0	3.0	1.0
**PONS**				
PnO	16.0	13.0	13.3	6.3
PnC	13.0	2.0	4.0	7.5
SubCD	0.0	0.0	5.7	7.3
DpMe	2.7	10.7	0.0	0.0
MVPO	0.0	0.0	1.3	0.3
LDTg	2.0	0.0	0.0	0.0
RtTg	6.0	0.0	0.0	0.0
SPTg	2.0	1.0	0.0	0.0
IRt	0.0	1.3	5.3	0.0
PCRt	0.0	0.0	0.7	2.3
PPTg	6.0	0.0	6.0	2.7
Tz	0.0	0.0	0.0	1.7
**MIDBRAIN**				
PaR	0.0	0.0	1.3	0.3
PBP	0.0	0.0	8.5	0.0
CIC	0.0	0.0	0.0	4.3
VLPAG	1.3	7.3	0.0	0.0

*The values show averaged counts per structure for each of the three animals in MLF and CVLM experiments. IPSI, ipsilateral to spinal injection; CONTRA, contralateral to spinal injection. 5n, motor trigeminal nucleus; 7n, facial nucleus; CIC, central nucleus of the inferior colliculus; Cu, cuneate nucleus; DpMe, deep mesencephalic nucleus; Gi, gigantocellular reticular nucleus; IO, inferior olive; IRt, intermediate reticular nucleus,; LDTg, laterodorsal tegmental nucleus; LPAG,lateral periaqueductal gray; LPGi, lateral paragigantocellular nucleus; LRt,lateral reticular nucleus; MdV, medullary reticular nucleus; Mlf, medial longitudinal fasciculus; MVe, medial vestibular nucleus, MVPO, medioventral periolivary nucleus; PaR, pararubral nucleus; PBP, parabrachial pigmented nucleus of the VTA; PCRt, parvicellular reticular nucleus; PnC, pontine reticular nucleus, caudal part; PnO, pontine reticular nucleus, oral part; PPTg, pedunculopontine tegmental nucleus; PPy, parapyramidal nucleus; RMg, raphe magnus nucleus; ROb, raphe obscurus nucleus, RPa, raphe pallidus nucleus; RR, retrorubral nucleus; RtTg, reticulotegmental nucleus; Sol, nucleus of the solitary tract; SPO, superior paraolivary nucleus; SPTg, subpeduncular tegmental nucleus; subCD, subcoeruleus nucleus; tz, trapezoid body; VLPAG, ventrolateral periaqueductal gray*.

### Medulla

**Figure 4:**
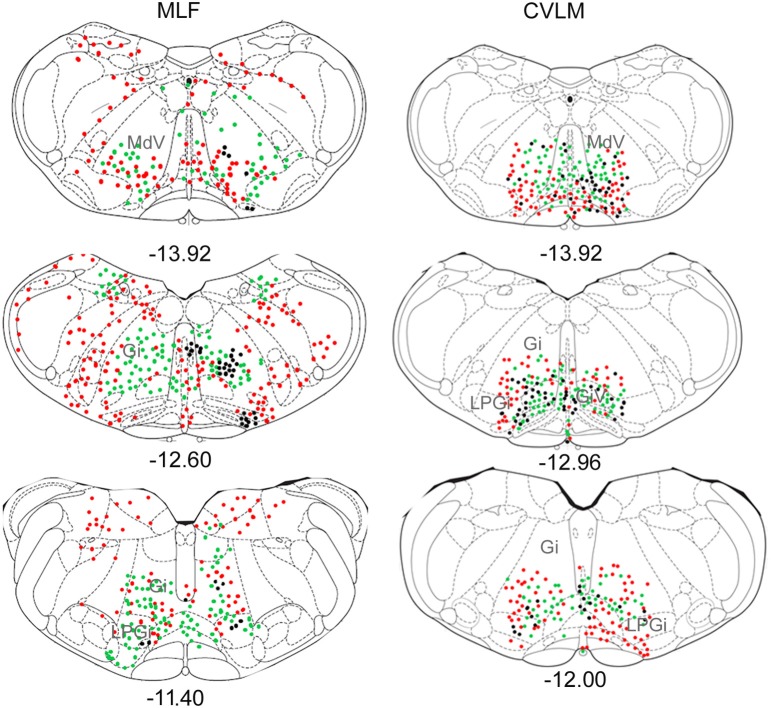
**Distribution of cells in medulla**. Outline diagrams of coronal sections through the medulla at three levels (anterior-posterior coordinates according to Bregma) showing the locations of cells following spinal injections in the right intermediate gray matter and injections within the MLF or right CVLM. Black circles represent double-labeled cells; green circles represent spinally-projecting cells; and red circles represent cells labeled from MLF or CVLM. Gi, gigantocellular reticular nucleus; LPGi, lateral paragigantocellular nucleus; MdD, medullary reticular nucleus.

Distributions of cells in the medulla for CVLM and MLF injections are shown in Figure 4. For MLF injections, the majority of double-labeled cells were found ipsilateral to the spinal injection (394 vs. 97 contralateral cells). The greatest numbers of double-labeled cells were found in the gigantocellular reticular nucleus (Gi), the inferior olivary complex (IO), medullary reticular nucleus, dorsal part (MdD), the MLF and the raphe magnus and obscurus (RMg, ROb). Small numbers of cells were noted in a variety of other structures (Table 3), including the nucleus of the solitary tract (sol) and the medial vestibular nucleus (MVe). Double-labeled cells following CVLM injections were also found predominantly ipsilateral to spinal injections (673 vs. 307 contralateral cells) and were present in many of the same structures observed for the MLF except that many more cells were located within the lateral paragigantocellular nucleus (LPGi) and the raphe pallidus (RPa) than were found for MLF injections. Additional cells were also present in sol and MVe along with the facial nucleus (7n) and the lateral reticular nucleus (LRt).

### Pons

**Figure 5:**
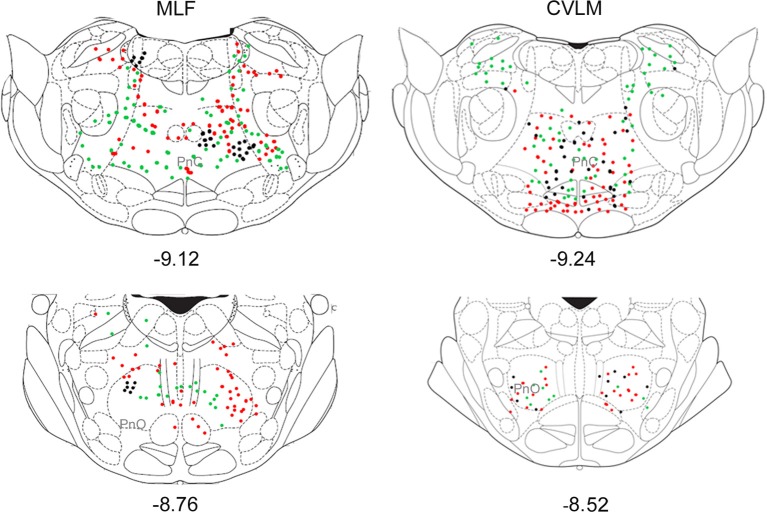
**Distribution of cells in pons**. Outline diagrams of coronal sections through the pons at two levels (anterior-posterior coordinates according to Bregma) showing the locations of cells following spinal injections in the right intermediate gray matter and injections within the MLF or right CVLM. Black circles represent double-labeled cells; green circles represent spinally-projecting cells; and red circles represent cells labeled from MLF or CVLM. PnC, pontine reticular nucleus, caudal part; PnO, pontine reticular nucleus, oral part.

Pontine cell distributions are shown in Figure 5. The majority of double-labeled cells in both experimental groups were found ipsilateral to spinal injections (143 vs. 84 for MLF and 109 vs. 84 for CVLM) and, in both groups, they were present in the oral and caudal part of the pontine reticular nucleus, (PnO and PnC). For MLF injections cells were also observed in the deep mesencephalic nucleus (DpMe) the reticulotegmental nucleus (RtTg) and the pedunculopontine tegmental nucleus (PPTg) and for CVLM injections cells were found in the dorsal subcoeruleus (subCD). Full details of other structures containing cells are given in Table 3.

### Midbrain

**Figure 6:**
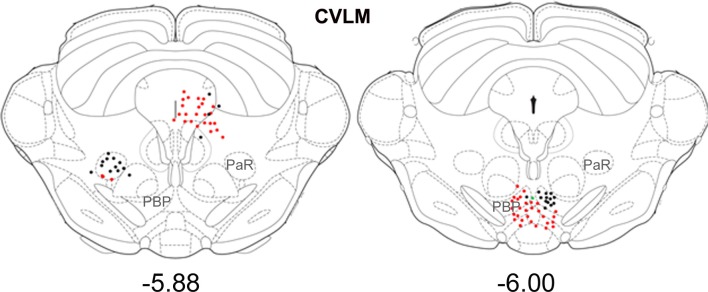
**Distribution of cells in midbrain**. Outline diagrams of coronal sections through the midbrain at two levels (Bregma anterior-posterior coordinates) showing the locations of cells following spinal injections and injections within the left CVLM. Black circles represent double-labeled cells; green circles represent spinally-projecting cells; and red circles represent cells labeled from CVLM. PaR, pararubral nucleus; PBP, parabrachial pigmented nucleus of the ventral tegmental area.

Small numbers of double-labeled cells were found in the midbrain (Figure 6). For CVLM injections, they were concentrated in two areas: the pararubral nucleus (PaR) and parabrachial pigmented nucleus of the ventral tegmental area (PbP). The only midbrain region containing cells following MLF injections was the ventrolateral periaqueductal gray (VLPAG).

### Axon terminals labeled from the CVLM

**Figure 7:**
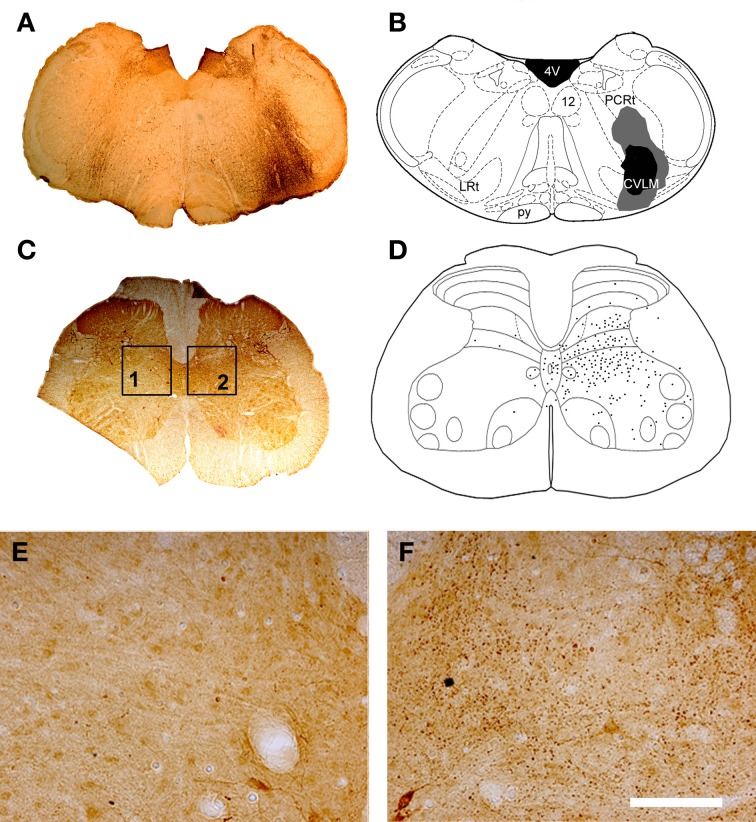
**CVLM injections and distribution of terminals in lumbar spinal cord. (A)** Shows a light micrograph of a transverse section through the medulla illustrating a CTb injection site. Injection sites were mapped onto outline diagrams of the medulla **(B)** obtained from the stereotaxic atlas of Paxinos and Watson (2005). The black area is the core of the injection and the gray area represents the spread of CTb. Plate **(C)** shows a transverse section through L4 that was reacted to reveal CTb-labeled terminals. **(D)** The distribution of terminals was mapped onto outline diagrams of the spinal cord. Note the predominance of terminals ipsilateral to the injection site which are concentrated in the intermediate gray matter. **(E,F)** Are magnified views of boxes 1 and 2 of plate **(C)**. Note the scarcity of terminals contralateral to the injection site **(E)** and the abundance of terminals on the ipsilateral side **(F)**. Scale bar **E** and **F** = 100 μm. 4V, 4th ventricle; 12, hypoglossal nucleus; CVLM, caudal ventrolateral medulla; LRt, lateral reticular nucleus; PCRt, parvicellular reticular nucleus; py, pyramid.

CVLM Injection sites (Figures 7A,B) were similar to those shown above for double-labeling experiments. Axon terminals were found throughout lumbar segments principally ipsilateral to the injection site (Figures 7C–F). The majority of terminals were concentrated within the intermediate gray matter (laminae V, VI, VII, and X). Few terminals were present in the dorsal horn above lamina V and in the ventral horn including motor nuclei and lamina VIII.

### Transmitter phenotypes

**Figure 8:**
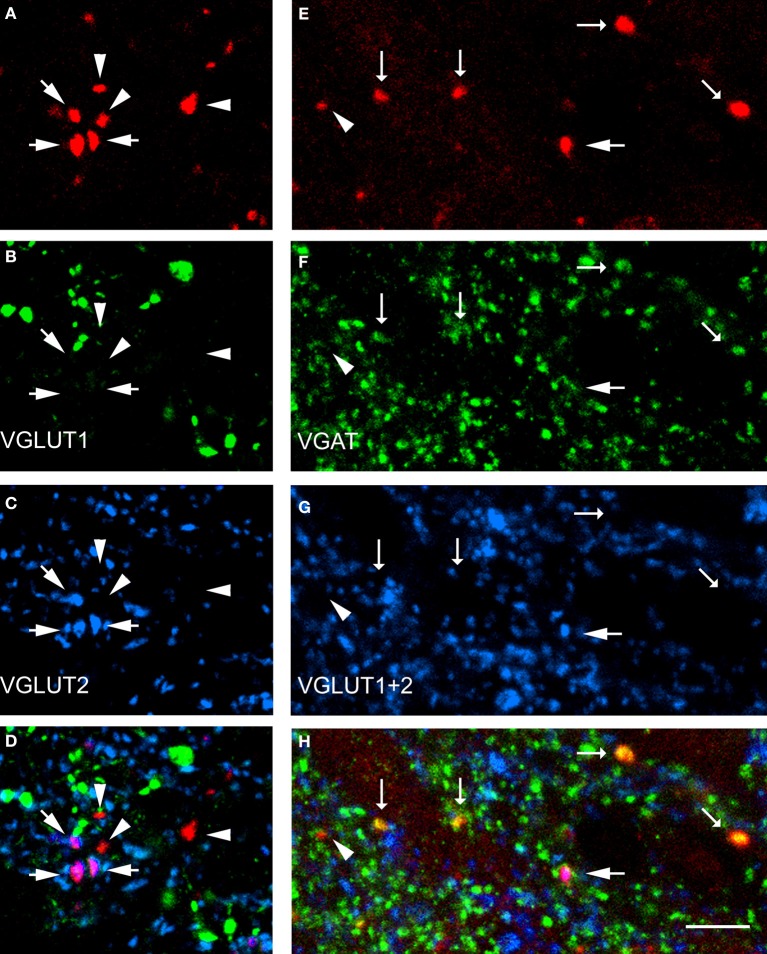
**Transmitter phenotypes of spinal axons projecting from the CVLM. (A–D)** Single optical sections of **(A)** CTb labeled terminals in the same optical plane for vesicular glutamate transporters (VGLUT)1 and 2 **(B,C)** and a merged image **(D)**. Arrows indicate VGLUT2-immunoreactive terminals and arrow heads indicate terminals unlabeled for both markers. **(E–H)** A similar series where tissue was reacted with a mixture of VGLUT1 and 2 antibodies and the vesicular GABA transporter (VGAT; **F,G**). Large arrows indicate VGLUT-immunoreactive terminals, small arrows indicate VGAT-immuno- reactive terminals and the arrowhead indicates a terminal that is not immunoreactive for either marker. Scale bar = 10 μm.

**Figure 9:**
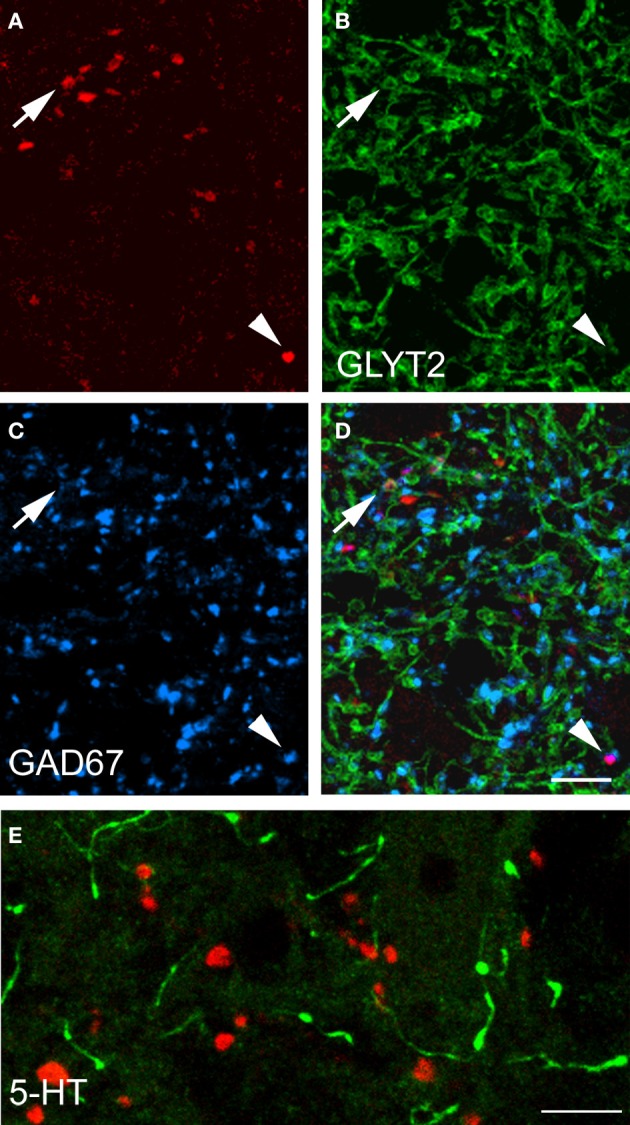
**Transmitter phenotypes of spinal axons projecting through the CVLM. (A–D)** Single optical sections of **(A)** CTb labeled terminals shown in the same plane for immunoreactivity for the glycine transporter T2 (GLYT2) and the 67 isoform of glutamate decarboxylase (GAD67; **B,C**) A merged image is shown in **(D)**. The arrow indicates a GLYT2-immuno- reactive terminal and the arrowhead is a GAD67 labeled terminal. **(E)** Shows immunoreactivity for serotonin (5-HT). Note the absence of 5-HT immunoreactivity (green) in CTb-labeled axon terminals (red). Scale bars = 10 μm.

Data concerning the immunoreactivity of terminals in L3, 4, and 5 which were labeled from the CVLM are given in Table 4. The majority of terminals were found to contain VGLUT2 (average ± *SD* 62.2 ± 3.13%). Figures 8A–D shows examples of terminals in the intermediate gray matter that are immunoreactive for VGLUT2 but not VGLUT1. Figures 8E–H also shows that some terminals are not immunoreactive for either VGLUT1 or VGLUT2 but contain VGAT (22.7 ± 2.2%). Further investigation of inhibitory terminals (Figures 9A–D) showed that they consisted of three types: (1) those immunoreactive for GLYT2 (12.5 ± 1.5%); (2) those immunoreactive for GAD 67 (8.7 ± 0.3%); (3) those that contained both markers (2.9 ± 1.0%). In addition a substantial number of terminals were not immunoreactive for VGLUT or VGAT (approximately 12%). None of these terminals were immunoreactive for serotonin (Figure 9E).

**Table 4:** **Percentages of immunoreactive terminals in the lumbar spinal cord labeled by CVLM injections**.

**Animal**	**No. terminals**	**VGLUT1**		**VGLUT2**
1	1042	0.38		64.59
2	903	1.44		63.34
3	936	0.64		58.65
Mean%		**0.82**		**62.20**
±*SD*		0.55		3.13
**Animal**	**No. terminals**	**VGLUT1+2**		**VGAT**
1	334	65.37		23.13
2	997	72.34		20.47
3	288	57.01		24.71
Mean%		**64.91**		**22.77**
±*SD*		7.68		2.15
**Animal**	**No. terminals**	**GLYT2**	**GAD67**	**GLY/GAD**
1	1220	11.30	8.85	2.77
2	1818	12.11	8.33	2.02
3	885	14.13	8.83	3.92
Mean%		**12.51**	**8.67**	**2.91**
±*SD*	–	1.46	0.29	0.96

*The table shows the total number of terminals analyzed per animal for each of the experiments, the percentages of terminals immunoreactive for each of the markers tested and the mean percentage (shown in bold) ± the standard deviation for each marker. VGLUT1, vesicular glutamate transporter 1; VGLUT2, vesicular glutamate transporter 2; VGAT, vesicular GABA transporter; GLYT2, glycine transporter 2; GAD67, 67 isoform of glutamate decarboxylase. VGLUT1+2, tissue incubated in a mixture of both antibodies*.

## Discussion

In this study we have shown that the cells that form pathways from the brainstem to the lumbar spinal cord passing through the MLF and CVLM, for the most part, have overlapping spatial distributions. The vast majority of cells in both pathways originate from reticular areas of the brainstem such as Gi, LPGi, and MdV in the medulla and PnO and PnC in the pons. In addition, both pathways contain raphe-spinal neurons and spinally projecting cells located within the inferior olivary complex. Double-labeled cells were found ipsilateral and contralateral to spinal injection sites, but there was a tendency for greater numbers of cells to be located ipsilaterally. Therefore both pathways contain a mixture of crossed and uncrossed axonal projections. Differences between the two pathways are subtle; the CVLM pathway projects predominantly to the ipsilateral gray matter of the spinal cord whereas a proportion of BS axons which pass through the MLF innervate both sides of the spinal gray matter (Nyberg-Hansen, 1965; Peterson et al., 1975; Martin et al., 1985; Mitani et al., 1988; Matsuyama et al., 1993, 1999, 2004). The most significant difference is that the CVLM pathway has few terminations in the ventral horn whereas many MLF fibers terminate within motor nuclei and are often concentrated within lamina VIII (Jones and Yang, 1985; Matsuyama et al., 2004; Du Beau et al., 2012).

### Technical considerations

The b subunit of cholera toxin has been used extensively as a retrograde tracer (Chen and Aston-Jones, 1995) but is transported in the anterograde as well as the retrograde direction (Ericson and Blomqvist, 1988). In common with other tracers, it is taken up by axons of passage in the CNS (Chen and Aston-Jones, 1995) and we have exploited this property to label cells whose axons pass through the CVLM and MLF in order to determine the origin of spinally-projecting cells with axons in the two pathways. However, it is almost impossible to label only one side of the MLF as a consequence of the inevitable spread of tracer across the midline and our sample of spinally-projecting MLF cells is likely to be composed of cells with axons that travel through left or right sides of the MLF whereas CVLM double-labeled cells will be exclusively unilateral with axons passing through the right CVLM only.

To compensate for variation between injection sites we attempted to identify consistent patterns of cell distribution for the three animals within each group quantitatively by averaging the numbers of double-labeled cells found within each structure and thus extracted the principal structures associated with spinally-projecting cells. Borders of many brainstem structures are not well-defined and therefore some cells may have been wrongly allocated by the mapping procedure we used which depended upon superimposing stylized diagrams (Paxinos and Watson, 2005) upon coronal sections at various levels of the brainstem. For example cells attributed to the mlf may have belonged to ROb or Gi. Another complication was that as we were injecting CTb into the brainstem itself, the area in the immediate vicinity of the injection site could not be used to identify double-labeled cells. This is possibly why no cells could be identified within RPa following MLF injections.

We also used the CTb tracing technique to label axon terminals in lumbar segments that originate or pass through the CVLM. The merits of this technique were discussed in a previous publication (Du Beau et al., 2012). A potential problem with the CTb method, however is that it may not label unmyelinated axons anterogradely. This could be the explanation of why we saw many cells in raphe nuclei following retrograde labeling but no serotonin immunoreactive terminals following anterograde labeling (see Du Beau et al., 2012 for a fuller Discussion). Finally, in addition to anterograde labeling of descending axons, CTb injections in the CVLM also label spinomedullary neurons retrogradely; hence some of the terminals we observed could have arisen from collateral axons of these cells. However, similar numbers of spinomedullary cells are labeled on both sides of the cord but very few terminals are present in the contralateral gray matter following CVLM injections which contrasts with the large numbers of terminals found in the ipsilateral gray matter. For this reason we consider it unlikely that our sample of terminals was contaminated by significant numbers of terminals from collateral axons of ascending neurons.

### Comparison with other studies

Previous studies have used lesion techniques, spinal injections of tracer substances or combinations of tract-tracing and spinal lesions to determine anatomical locations of cells of origin of bulbospinally-projecting pathways in a variety of species including rat (Leong et al., 1984; Zemlan et al., 1984; Jones and Yang, 1985; Rye et al., 1988; Reed et al., 2008), mouse (Terashima et al., 1984; VanderHorst and Ulfhake, 2006) cat (Torvik and Brodal, 1957; Kuypers and Maisky, 1975; Basbaum and Fields, 1979; Mitani et al., 1988), opossum (Martin et al., 1979) and monkey (Kneisley et al., 1978). Studies of brainstem cells following lumbar injections in the rat (Leong et al., 1984; Reed et al., 2008) show similar distribution patterns to the Fluorogold-labeled cells documented in the present study. An unexpected finding was that a major component of both pathways appears to originate from cells within the inferior olivary complex. Although the older literature often refers to the olivo-spinal tract, the existence of this pathway has been a matter of contention (e.g., see Brodal et al., 1950; cf Voneida, 1967). Nevertheless, Leong et al. (1984) also reported the presence of spinally projecting cells within the IO and it remains an open question as to whether the pathway exists. Zemlan et al. (1984) observed large clusters of spinally-projecting cells just dorsal to the IO in rats following injections of HRP into T10. As it is difficult to define the exact borders of brainstem nuclei in many cases (see Technical issues above) it is possible that some of the cells we plotted as IO cells belong to adjacent reticular nuclei and were misattributed or perhaps even misplaced anatomically (see Figure 2A).

In addition to reticulospinal components mentioned above, the other major components of these pathways originated from raphe nuclei; an observation which is consistent with many previous reports (e.g., see Bowker et al., 1981). Small numbers of cells were also noted within structures previously reported to have projections to the spinal cord including the nucleus of the solitary tract (Loewy and Burton, 1978), the medial vestibular nucleus (Bankoul and Neuhuber, 1992) the sub coeruleus, which is a source of noradrenergic fibers (Kuypers and Maisky, 1975; Hancock and Fougerousse, 1976; VanderHorst and Ulfhake, 2006) and the parabrachial pigmented nucleus of the ventral tegmental area (Hancock and Fougerousse, 1976), which is likely to be a source of dopaminergic fibers (Hasue and Shammah-Lagnado, 2002; VanderHorst and Ulfhake, 2006).

### Comparison of findings for MLF and CVLM

Not only do the major elements of the MLF and CVLM pathways originate principally from similar brainstem structures but both pathways have similar proportions of excitatory and inhibitory components (Du Beau et al., 2012). The majority of axon terminals in both pathways contain VGLUT2 and thus are mostly excitatory (58 and 62%; MLF and CVLM respectively) but there is also a significant inhibitory component in both pathways that consists of purely GABAergic terminals (7 and 9%), purely glycinergic terminals (9 and 13%) and a small group that are both GABAergic and glycinergic (3 and 3%). Components of the BS pathways are found in the ventromedial, dorsolateral and ventrolateral white matter of the spinal cord and there is evidence that these different pathways originate from distinct regions of the brainstem and may be highly ordered (Nyberg-Hansen, 1965; Kuypers and Maisky, 1977; Basbaum et al., 1978; Jones and Yang, 1985; Martin et al., 1985; Mitani et al., 1988; VanderHorst and Ulfhake, 2006). Our study indicates that the MLF and CVLM pathways contain axons of cells originating from both pontine and medullary locations. According to the atlas of Paxinos and Watson (2005) the MLF begins to form its medial and lateral components at roughly −5.7 mm anterior-posterior to the interaural zero point. As our CVLM injections were made at −4.8 mm it is unlikely that lateral components of the MLF pathway were labeled and therefore axons passing through the CVLM presumably form an additional pathway (see also Mitani et al., 1988). However, we cannot exclude the possibility that some of the double-labeled cells we observed give rise to bifurcating axons which pass through both structures.

### Functional implications

Bulbospinal pathways are known to have monosynaptic actions on motoneurons and premotor interneurons (Grillner and Lund, 1968; Floeter et al., 1993; Gossard et al., 1996; Bannatyne et al., 2003; Jankowska et al., 2003; Riddle et al., 2009; Galea et al., 2010) and they also have profound influences on sensory systems (especially nociceptive pathways), respiration and autonomic activity (Basbaum and Fields, 1979; Hardy et al., 1998; Tavares and Lima, 2002). These pathways form extensive termination patterns within the spinal gray matter. Individual SB cells can have axons that innervate several segmental levels (Huisman et al., 1981; Cavada et al., 1984; Matsuyama et al., 2004; Reed et al., 2008) and provide input to extensive areas of the gray matter, with some axon collaterals projecting to both sides of the cord (Nyberg-Hansen, 1965; Peterson et al., 1975; Martin et al., 1985; Holstege, 1996; Matsuyama et al., 2004). Therefore these systems appear to be ideally suited to coordinate activity of diverse neuronal networks on both sides of the cord located at different segmental levels. They also contain a multiplicity of neurotransmitters and neuromodulators, including inhibitory and excitatory amino acids, monoamines and peptides (Holstege, 1996; Grillner et al., 2000; VanderHorst and Ulfhake, 2006; Jordan et al., 2008) and thus have the capacity to facilitate or depress network activity via direct and indirect inhibitory and excitatory synaptic actions on spinal neurons along with modulatory effects.

The moot question is what is the function of the CVLM pathway given its apparent similarities to the MLF pathway? A clue to the function of this pathway is provided by its anatomical organization: generally it innervates a more circumscribed region of the midlumbar spinal gray matter than the MLF pathway and, unlike the MLF pathway, does not have many terminations in motor nuclei and lamina VIII. Also very few axons passing through the CVLM cross to form terminals in the contralateral gray matter and therefore the influence of this lateral pathway appears to be more restricted than the MLF pathway. In a study of CVLM terminations in the thoracic spinal cord, Hardy et al. (1998) reported that in addition to the ipsilateral pathway, which terminates principally on sympathetic preganglionic neurons, there is a contralateral component that specifically targets phrenic motoneurons. These authors suggested that a possible function of the CVLM pathway is to coordinate respiratory and autonomic function. In midlumbar segments, CVLM axons terminate predominantly in the deep dorsal horn and intermediate gray matter and are ideally located to influence premotor interneurons that are also found in this region (Coulon et al., 2011; Tripodi et al., 2011). Thus the function of the CVLM pathway may be to harmonize motor activity with respiratory and autonomic activity to produce the coordinated output of these systems required for physical exercise.

### Conflict of interest statement

The authors declare that the research was conducted in the absence of any commercial or financial relationships that could be construed as a potential conflict of interest.

## Anatomical Abbreviations

4v: 4th ventricle
5n: motor trigeminal nucleus
7n: facial nucleus
12n: hypoglossal nucleus
CIC: central nucleus of the inferior colliculus
Cu: cuneate nucleus
CVLM: caudal ventrolateral medulla
DpMe: deep mesencephalic nucleus
Gi: gigantocellular reticular nucleus
Gr: gracile nucleus
IO: inferior olive
IRt: intermediate reticular nucleus
LDTg: laterodorsal tegmental nucleus
LPAG: lateral periaqueductal gray
LPGi: lateral paragigantocellular nucleus
LRt: lateral reticular nucleus
MdV: medullary reticular nucleus
Mlf: medial longitudinal fasciculus
MVe: medial vestibular nucleus
MVPO: medioventral periolivary nucleus
PaR: pararubral nucleus
PBP: parabrachial pigmented nucleus of the VTA
PCRt: parvicellular reticular nucleus
PnC: pontine reticular nucleus, caudal part
PnO: pontine reticular nucleus, oral part
PPTg: pedunculopontine tegmental nucleus
PPy: parapyramidal nucleus
Py: pyramid
RMg: raphe magnus nucleus
Rob: raphe obscurus nucleus
RPa: raphe pallidus nucleus
RR: retrorubral nucleus
RtTg: reticulotegmental nucleus
Sol: nucleus of the solitary tract
SPO: superior paraolivary nucleus
SPTg: subpeduncular tegmental nucleus
subCD: subcoeruleus nucleus
tz: trapezoid body
VLPAG: ventrolateral periaqueductal gray.

**Other Abbreviations**
CTb: cholera toxin B subunit
CST: DAB 3,3′-diaminobenzidine
GAD-67: glutamic acid decarboxylase 67 isoform
GLY T2: glycine transporter 2
HRP: horseradish peroxidise
IgG: immunoglobulin gamma
PB: Phosphate buffer
PBS: Phosphate buffer saline
PBST: phosphate buffer saline containing 0.3% Trition X-100
RST: reticulospinal tract
SD: standard deviation
VGAT: vesicular GABA transporter
VGLUT: vesicular glutamate transporter.
